# Stability of Unicortical versus Bicortical Metacarpal Fracture Internal Fixation Trial (SUBMIT): study protocol for a randomized controlled trial

**DOI:** 10.1186/s13063-016-1538-3

**Published:** 2016-08-18

**Authors:** Feiran Wu, Katie Young, Mohammad Shahid, Peter Nightingale, Surabhi Choudhary, Michael Craigen, Rajive Jose, Mark Foster

**Affiliations:** 1 The Royal Orthopaedic Hospital, Bristol Road South, Birmingham, B31 2AP UK; 2 Queen Elizabeth Hospital, University Hospitals Birmingham, Metchley Lane, Birmingham, B15 2TH UK; 3 Queen’s Medical Centre, Derby Road, Nottingham, NG7 2UH UK

**Keywords:** Bicortical, Metacarpal fractures, Unicortical

## Abstract

**Background:**

Metacarpal fractures are common, accounting for 40 % of all hand injuries. The use of plates for the fixation of these fractures allows early aggressive hand therapy post-operatively, reducing post-operative stiffness. Traditionally, bicortical fixation is the standard practice, where both dorsal and palmar cortices of the metacarpal are drilled through, with screws engaging both cortices. Recent biomechanical studies have shown that unicortical fixation, where only the near cortex is drilled and engaged by the screw, results in no difference in stiffness, load to failure or failure mechanism, when compared with bicortical fixation. This trial aims to compare fracture union, complication rate and functional outcomes between unicortical and bicortical fixation for adults with displaced metacarpal fractures.

**Methods/Design:**

All adults with displaced diaphyseal metacarpal fracture requiring plate fixation are potentially eligible to take part in this study. A total of 315 consenting patients will be randomly allocated to either unicortical or bicortical plate and screw fixation. The surgery will be performed in specialist hand trauma units across the UK. Data regarding fracture healing, hand function, quality of life, and complications will be collected at 2 weeks, 6 weeks and 6 months following surgery.

**Discussion:**

This pragmatic, prospective, multi-centre, randomized controlled trial is expected to deliver results in 2018.

**Trial registration:**

ISRCTN 18006607. Registered on 19 Nov 2015.

## Background

### Background and rationale

Metacarpal fractures are common, accounting for 40 % of all hand injuries; many can be treated non-operatively [1]. Surgery is reserved for cases in which an adequate reduction of both angular and rotational deformity cannot be maintained or where an adjacent ray is damaged. Metacarpal shaft fractures may be transverse, where the deformity is typically apex–dorsal. Fractures may also be spiral or oblique; these are more unstable, and reduction must restore rotational alignment as a first priority.

A variety of surgical strategies exist, including percutaneous Kirschner wiring, intramedullary fixation, and osteosynthesis with plate and screw construction [1]. The use of plates for the fixation of metacarpal fractures was first documented by Burton and Eudell in 1958 and became widespread in the 1970s and 1980s [2]. A plate secured along the dorsal midline of the metacarpal has been shown to be the best biomechanical method of fixation, with significantly more stability than Kirschner wiring or intramedullary fixation [3]. As a result, metacarpal plating allows earlier aggressive hand therapy post-operatively than alternative treatment methods, reducing post-operative stiffness. This is now the standard of care for unstable or displaced diaphyseal metacarpal fractures.

Traditionally, bicortical fixation is the standard practice, where both dorsal and palmar cortices of the metacarpal are drilled though and the screws engage both cortices. However, such practice is not without risk. In this method, the flexor tendons and neurovascular bundles are at risk from over-zealous drilling through the palmar cortex. Correct screw size selection is critical, as overly long screws can irritate and cause rupture of the flexor tendon [4]. More recently, the biomechanical superiority of bicortical fixation over unicortical fixation in metacarpal fractures has been questioned, as the predominant force acting on the metacarpal is apex–dorsal loading (bending in a palmar direction), owing to the action of the flexor tendons [5]. Unicortical fixation is a surgically less complex operation, can theoretically cause less damage to surrounding soft tissues and avoids the complications associated with incorrectly sized screws. This is currently the standard treatment for the fixation of maxillofacial fractures [6].

There is a paucity in the literature of studies comparing unicortical and bicortical internal fixation of fractures in the hand, with no clinical or *in-vivo* evidence. In cadaveric studies, Dona *et al*. [5] showed that there was no difference in the stiffness, load to failure or failure mechanism between unicortical and bicortical fixation of fractures in 18 freshly frozen human metacarpals. The mean load to failure was 596 N for the unicortical group and 541 N for the bicortical group, using a four-point bending protocol [5]. Afshar *et al*. [7] showed that bicortical fixation had a load to failure one-fifth greater than unicortical fixation in 20 cadaveric human metacarpals, with a mean load to failure of 370 N for unicortical fixation and 450 N for bicortical fixation, using cyclic loading. However, Afshar *et al*. [7] did not take into account the biomechanical advantage of an intact soft tissue envelope and conceded that they could not correlate their findings with the loads experienced by the patient during rehabilitation following surgical fixation [7]. Khalid *et al*. [8] showed that bicortical fixation resulted in higher pull-out strengths in 40 cadaveric human proximal phalanges, but recommended unicortical fixation for diaphyseal fractures, as the pull-out force far exceeds that generated by the flexor tendon in passive and active finger flexion [8]. In an animal fracture model, Ochman *et al*. [9] found that the stability of unicortical and bicortical locking and nonlocking plate fixation differed, with the maximum load to failure greater in locking fixation methods [9]. Locking plates can create unique problems in the hand, however. The stability of conventional bone plating systems is achieved when the head of the screw compresses the fixation plate to the bone as the screw is tightened, generating a precisely contoured fit. Locking plate and screw systems circumvent the need for precise plate adaptation and achieve stability through a device that ‘locks’ the screw to the plate while the screw shaft secures the bone [10]. Imprecisely contoured plates that are offset from the bone can interfere with the extensor mechanism, inhibiting tendon glide and causing bursa formation [9].

### Objectives

The primary objective is to compare union rates between unicortical and bicortical screw and plate fixation of diaphyseal metacarpal fractures. Patients will be assessed for fracture union 6 months after their fracture fixation using radiographs, assessed by an independent musculoskeletal radiologist and a senior consultant hand surgeon; a third consultant hand surgeon will be consulted for verification in cases where there is disagreement.

The secondary objectives are to compare the implant failure rate, complication rate and functional outcomes between the two groups. Functional outcome measures will include scores on the Disabilities of the Arm, Shoulder and Hand Outcome Measure, the Patient Evaluation Measure, the EuroQol five dimensions questionnaire, and a visual analogue scale for pain, function, movement and satisfaction, as well as a range-of-motion assessment of the affected wrist and finger.

## Methods/Design

### Design

This is a prospective, pragmatic, multi-centre, randomized controlled non-inferiority trial. This study was approved by the NHS Research and Ethics Committee (no 14/WM/1212, 12 December 2014) and the UK National Health Service (NHS) Coordinated System for Gaining NHS Permission (National Institutes for Health Research Clinical Research Network study ID 18642).

### Study setting

This study has commenced recruitment at University Hospitals Birmingham. Other specialist hand units including University Hospitals Coventry and Warwick and Queen Victoria Hospital, East Grinstead will aim to start recruitment in 2016.

### Eligibility criteria

Patients will be eligible for this study if they meet the following criteria:

- Male or female, aged 18 years or above and able to give informed consent
- Diagnosed with metacarpal diaphyseal, extra-articular fractures
- The treating surgeon believes that they would benefit from operative fixation of the fracture
- Suitable for regional axillary brachial plexus anaesthesia or general anaesthesia
- Surgical fixation will be within 10 days of injury

Patients will be excluded from participation in this study if any of the following apply:

- Pregnant or intend to be pregnant in the next 6 months
- Non-osteoporotic pathological fracture or a previous fracture of the same metacarpal
- Other non-metacarpal injury to the same upper limb requiring surgery
- Major nerve injury (e.g., median, ulnar or radial), blood vessel or tendon injury
- Multi-trauma patient
- Revision procedure i.e. previous fracture of  the same metacarpal
- Known malignancy
- There is evidence that the patient would be unable to adhere to trial procedures or complete questionnaires, e.g. because of cognitive impairment or intravenous drug abuse

### Recruitment, ethics and consent

Patients will be recruited from NHS clinics and accident and emergency departments of at least three teaching hospitals in England, which each have a specialist hand surgery unit. A member of the surgical team will introduce the trial to eligible patients; if patients would like more information, they will be given the trial patient information documentation and a member of the research team will be informed. Patients will usually have 24 hours to consider participation but will have a minimum of 60 min in exceptional circumstances (e.g. trauma theatre space available on the day of presentation). Written consent will be obtained by a member of the research team, which will include consent for the randomized data of the study to be published in scientific peer-reviewed journals. Baseline pre-operative assessments will be obtained following consent. Randomization will take place immediately before surgery.

### Trial interventions

All of the hospitals involved in this trial currently use both of the methods of fixation and all of the surgeons involved will be familiar with both techniques. Operative fixation of fractures will take place under an axillary brachial plexus regional anaesthetic block or a general anaesthetic block, according to the anaesthetist’s preference. Patients will be randomized immediately pre-operatively to the method of fixation using a trial management database program entitled ‘Clinical Research Tool’ (CREST), designed by University Hospitals Birmingham computer and information technology developers and the trial statistician. Patients will be blinded to their treatment throughout the trial duration.

In this trial, the details of the surgery will be left to the discretion of the surgeon to ensure that the results of the trial maintain external validity.

#### Fixation method

A 2.0 mm straight plate will be applied through an incision over the dorsal aspect of the hand. The details of the surgical approach will be left to the discretion of the surgeon. A minimum of two screws will be used on each side of the fracture to hold the fracture. For patients assigned to unicortical fixation, only the near cortex will be drilled and nonlocking screws only engaging the near cortex will be used to hold the reduction. For patients assigned to bicortical fixation, both the near and far cortices will be drilled and nonlocking screws will engage both cortices. The same type of plate and screws will be used for both fixation methods.

Patients will be blinded to their treatment regardless of the type of anaesthetic used. For patients undergoing a regional anaesthetic block, a visual drape will be used and audio-visual distraction will be available.

The method of closure will be left to the discretion of the surgeon. Both patient groups will have a bulky dressing following the operation; the dressing will not inhibit the movement of the fingers and will allow early controlled range-of-movement exercises.

### Participant timeline

**Fig. 1:**
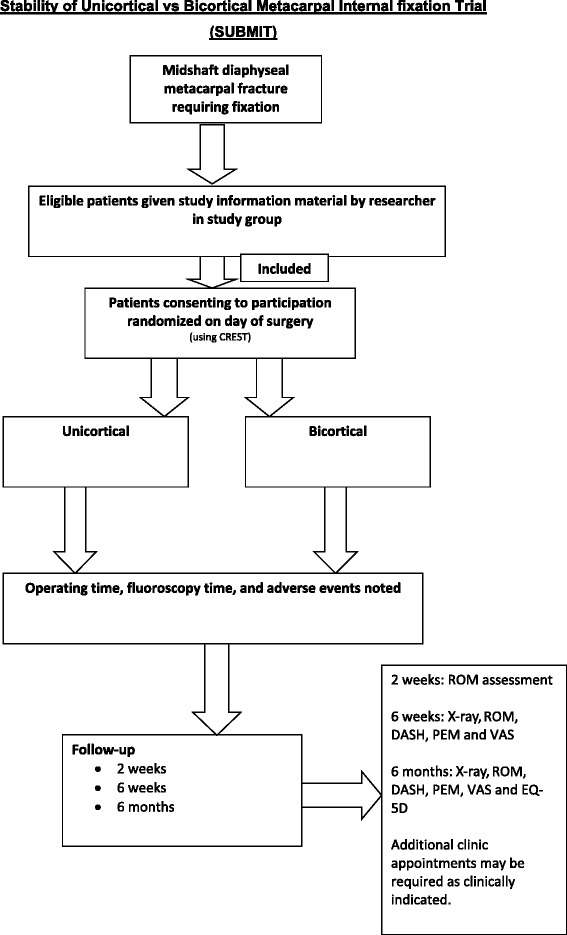
Participant timeline. CREST, Clinical Research Tool; DASH, Disabilities of Arm, Shoulder and Hand Outcome Measure; EQ-5D, EuroQol five dimensions questionnaire; PEM, Patient Evaluation Measure; ROM, range of motion; VAS, visual analogue scale.

Patients randomized into the two groups will receive standardized, written hand therapy advice detailing the exercises they need to perform for rehabilitation following their injuries. All participating patients in both groups will be advised to move their wrist and finger joints within comfort limits. Dressings will be removed after between 5 and 7 days in a dressings clinic. Two weeks after surgery, patients will be assessed by a hand therapist and begin formal, standardized, active range-of-movement exercises according to a pre-defined protocol. Hand therapists will be blinded to the method of fixation. Rehabilitation for both treatment groups will be the same. A record of any additional rehabilitation input (type of input and number of additional appointments), together with a record of any other investigations or interventions, will be requested as part of the 2 week, 6 week and 6 month clinical follow-ups and this will also form part of the trial dataset. Clinicians will assess the patients at 6 weeks and 6 months post-operatively and will not be blinded for the purpose of assessing radiographs for complications (Fig. 1).

Techniques common in long-term cohort studies will be used to ensure minimum loss to follow-up, such as collection of several contact addresses, telephone numbers and email addresses. Considerable efforts will be made by the trial team to keep in touch with patients throughout the trial by means of newsletters, telephone calls, mobile text messages and letters to ensure attendance.

### Outcome measures

Patient characteristics and baseline (pre-injury) functional status will be collected after consent to take part in the trial has been obtained. Structured information regarding other injuries that may affect outcome, e.g. previous wrist injury, will be collected. All patients will be included in the analysis. Assessments will be performed at 2 weeks, 6 weeks and 6 months following patients’ operation. Results will be collected by a blinded hand therapist.

#### Primary outcome measure

The primary outcome measure for this study will be assessment of fracture union at 6 months.

Three-view (anteroposterior, lateral, and oblique views) radiographs of the hand at admission, 6 weeks and 6 months will be independently evaluated by two observers (a consultant musculoskeletal radiologist and an orthopaedic hand surgeon). In situations where there is conflict, a third senior hand surgeon will be asked to assess the radiographs and cast a deciding vote. The initial fracture assessment will include fracture displacement and angulation measured on a standard Picture Archiving and Communication System workstation (Agfa Impax 6, Agfa Healthcare), and pre-existing osteoarthritis in the hand. Union assessment on post-operative radiographs will be based on visibility of fracture lucency on each view. Fracture union will be defined as ‘united’ if trabeculae are seen crossing sclerosis at the fracture, ‘partially united’ if part of a lucent fracture line is still seen, ‘probably united’ if there is no clear gap but the fracture cannot be defined as ‘united’ based on the criteria, and ‘not united’ if fracture lucency can be clearly seen. If the fracture is partially united, the ratio of width of bridging trabeculae to the entire fracture length will also be recorded as an objective measure of degree of fracture union. Assessment of surgical plate and screws will be recorded for lucency at the metal bone interface, migration or breakage.

#### Secondary outcome measures

##### Intra-operative parameters

Surgical time and fluoroscopy time will be recorded for each patient.

##### Disabilities of Arm, Shoulder and Hand Outcome Measure

This is a 30-item, self-reported questionnaire designed to provide a more general measure of physical function and symptoms in people with musculoskeletal disorders of the upper limb [11].

##### The Patient Evaluation Measure

This consists of 11 self-reported questions relating to subjective hand function, scored on a Likert scale of 1 to 7. Symptoms assessed are feeling, cold intolerance, pain, dexterity, wrist movement, subjective grip strength, daily activities, work appearance and a general assessment of wrist and hand function [12].

##### The EuroQol five dimensions questionnaire

This is a validated, generalized, quality-of-life questionnaire consisting of five domains related to daily activities, with a five-level answer possibility. The combination of answers leads to the quality-of-life score [13].

##### Visual analogue scale scores

These will be obtained for pain, movement, function and satisfaction based on a 0–100 scale.

##### Complications

All complications will be recorded.

##### Radiographic evaluation

Standard posterior-anterior, oblique and lateral radiographs will be taken at 6 weeks and 12 months after the procedure.

##### Stiffness

The Total Active Motion score, composite flexion, and wrist movement will be measured at each follow-up visit to assess range of motion.

### Sample size

Published data indicate a non-union rate of 8 % when plates and screws are used to fix metacarpal fractures [14]. Assuming this value in each group, a total of 252 patients (126 patients per group) would have an 80 % chance of demonstrating equivalence, with a margin of 10 %, of the failure rates of unicortical and bicortical fixation with 95 % confidence. From previous local audits of hand trauma studies, a drop-out rate of 20 % can be expected. Assuming a drop-out rate of 20 %, we would need 315 patients in total.

The level of 10 % is based on the limited data available currently on what differences could be expected after treating metacarpal fractures with bicortical fixation, compared with unicortical fixation.

### Patient allocation

After patients have provided baseline assessments and been checked for eligibility they will be asked for their informed consent to take part in the trial. The method of fixation will be allocated using a secure, centralized, NHS N3 computer database (CREST) via a secure web-browser page by a member of the research team. Randomization will be conducted on a 1:1 basis, stratified by centre. The allocation sequence will be based on an algorithm written by the trial statistician and the CREST developer.

Stratification by centre will help to ensure that any clustering effect related to the centre itself will be equally distributed in the trial arms.

### Blinding

Trial participants, hand therapists conducting the Total Active Motion assessment, composite flexion and wrist movements, and research nurses administering the Disabilities of Arm, Shoulder and Hand Outcome Measure, Patient Evaluation Measure, visual analogue scales and EuroQol five dimensions questionnaire will be blinded to the method of intervention.

The operating surgeon, clinician assessing for complications at follow-up visits and individuals assessing the patient radiographs will not be blinded.

Blinding will be achieved by only allowing the permissible individuals to have access to the method of fixation information, the operation record and radiographs of the participant.

### Data management

All trial data will be stored on the secure NHS N3 server based at the host hospital, i.e. University Hospitals Birmingham. The study team from the host hospital, from the University Hospitals Birmingham NHS Trust, or relevant regulatory authorities, where it is relevant to their taking part in this research, may have access to participants’ data. Such information will be treated as strictly confidential, and will be handled in accordance with the provisions of the UK Data Protection Act 1998.

### Statistical analysis

Baseline data (e.g. age and sex) will be summarized to check comparability between treatment arms, and to highlight any characteristic differences between those individuals in the study. Standard statistical summaries (e.g. medians and quartiles or means and standard deviations, dependent on the distribution of the outcome) and graphical plots will be presented for the primary and secondary outcome measures.

Differences between treatment groups will be assessed on an intention-to-treat basis. Tests will be two-sided and considered to provide evidence for a significant difference if *P* < 0.05 (5 % significance level). Estimates of treatment effects will be presented with 95 % confidence intervals.

The temporal patterns of any complications will be presented graphically and, if appropriate, a time-to-event analysis (Kaplan–Meier survival analysis) will be used to assess the overall risk and risks within individual classes of complications (e.g. implant failure).

Missing data are not expected to be a problem for this study. If the degree of data loss is relatively low, the primary analysis will be based on complete cases only, with analysis of imputed datasets used to assess the sensitivity of the analysis to the missing data. If the level of missing data is higher than expected, missing data will be imputed using the multiple imputation facilities available in R (http://www.r-project.org/).

### Organization

#### Monitoring

A data monitoring committee will review accumulating data and make recommendations to the trial steering committee with respect to trial conduct and participant safety. Charters outline the roles and responsibilities of these committees. The data monitoring and trial steering committees will be independent of the sponsor and will have no competing interests. Interim analysis and adverse event data will be performed by the trial statistician and made available to the data monitoring and trial steering committees.

A trial conduct audit will be conducted internally; results will be made available to the data monitoring and trial steering committees. Significant protocol amendments will be made to the NHS Research Ethics Committee, UK Clinical Research Network, ISRCTN trial registry and relevant journals where the protocol is published.

## Discussion

The SUBMIT trial is, to our knowledge, the first clinical trial to assess the efficacy of unicortical screw and plate fixation in patients with metacarpal fractures. Unicortical plate and screw fixation of metacarpal fractures is a less technically demanding and quicker procedure with, theoretically, less soft tissue disruption and potentially fewer complications.

Published studies have not proven a superiority of one fixation method over the other in cadaveric models. This study has been designed as a non-inferiority trial. We believe that should this study demonstrate equivalence between unicortical and bicortical fixation, unicortical fixation may become the standard method of fixing metacarpal fractures requiring surgery in the future.

## Trial status

Recruitment commenced in June 2015 and is expected to conclude in May 2018. Additional centres are expected to commence recruitment in 2016. The first results of the study are expected in late 2018.

## Abbreviations

CONSORT: Consolidated Standards of Reporting Trials
CREST: Clinical Research Tool
NHS: UK National Health Service
